# Design of a nanobiosystem with remote photothermal gene silencing in *Chlamydomonas reinhardtii* to increase lipid accumulation and production

**DOI:** 10.1186/s12934-023-02063-9

**Published:** 2023-03-31

**Authors:** Hossein Alishah Aratboni, Nahid Rafiei, Ashanti Concepción Uscanga-Palomeque, Itza Eloisa Luna Cruz, Roberto Parra-Saldivar, Jose Ruben Morones-Ramirez

**Affiliations:** 1 Universidad Autónoma de Nuevo León, UANL. Facultad de Ciencias Químicas, Av. Universidad S/N. CD. Universitaria, San Nicolás de los Garza, 66455 Nuevo León, México; 2 Centro de Investigación en Biotecnología Y Nanotecnología, Facultad de Ciencias Químicas, Parque de Investigación e Innovación Tecnológica, Universidad Autónoma de Nuevo León, Km. 10 Autopista Al Aeropuerto Internacional Mariano Escobedo, 66629 Apodaca, Nuevo León, México; 3 Department of Plant Production and Genetics, School of Agriculture, Shiraz University, Km. 12 Shiraz-Isfahan Highway, Bajgah Area, Shiraz, 71441-65186 Iran; 4 School of Engineering and Sciences, Tecnologico de Monterrey, Ave. Eugenio Garza Sada 2501, CP 64849 Monterrey, NL México; 5 Institute of Advanced Materials for Sustainable Manufacturing, Tecnologico de Monterrey, 64849 Monterrey, Nuevo Leon Mexico

**Keywords:** Gold nanoparticles, Photothermal, Optical switch, LED light source, Gene silencing, Lipid accumulation, Microalgae

## Abstract

Research development in the precise control of gene expression in plant cells is an emerging necessity that would lead to the elucidation of gene function in these biological systems. Conventional gene-interfering techniques, such as micro-RNA and short interfering RNA, have limitations in their ability to downregulate gene expression in plants within short time periods. However, nanotechnology provides a promising new avenue with new tools to overcome these challenges. Here, we show that functionalized gold nanoparticles, decorated with sense and antisense oligonucleotides (FANSAO), can serve as a remote-control optical switch for gene interference in photosynthetic plant cells. We demonstrate the potential of employing LEDs as optimal light sources to photothermally dehybridize the oligonucleotides on the surface of metallic nanostructures, consequently inducing regulation of gene expression in plant cells. We show the efficiency of metallic nanoparticles in absorbing light from an LED source and converting it to thermal energy, resulting in a local temperature increase on the surface of the gold nanoparticles. The antisense oligonucleotides are then released due to the opto-thermal heating of the nanobiosystem composed of the metallic nanoparticles and the sense-antisense oligonucleotides. By applying this approach, we silenced the *Carnitine Acyl Carnitine Translocase* genes at 90.7%, resulting in the accumulation of lipid bodies in microalgae cells. These results exhibit the feasibility of using functionalized gold nanoparticles with sense and antisense oligonucleotides to enhance nucleic acid delivery efficiency and, most importantly, allow for temporal control of gene silencing in plant cells. These nanobiosystems have broad applications in the development and biosynthesis of biofuels, pharmaceuticals, and specialized chemicals.

**Supplementary Information:**

The online version contains supplementary material available at 10.1186/s12934-023-02063-9.

## Introduction

Plant biotechnology plays a critical role in addressing global challenges in various industries, such as food, pharmaceutical, cosmetic, and renewable energy [1, 2]. Genetic engineering is a key tool for developing and altering metabolic pathways to enhance the synthesis of novel or valuable molecular compounds, including clinically relevant molecules, proteins, and lipids. Downregulation of gene expression is a commonly used genetic engineering technique, which can occur during either the level of transcription or post-transcription [3], and is widely used to control gene expression. Generally, downregulation of gene expression can be performed through gene silencing [4]; where micro-RNA (miRNA) and short interfering RNA (siRNA) can be used to activate and repress the genetic expression [5]. These short non-coding RNA molecules can exert their sequence-specific post-transcriptional gene silencing through cleavage of a target mRNA mediated by a small RNA–protein complex in an RNA-induced silencing complex (RISC) [6]. In addition to being a region of intense and preliminary research, gene silencing is a key technique in future technologies, especially those involved in the bioproduction of lipids. Increased production of lipids within plants is highly relevant since these compounds are the precursors of biofuels. In this work, we report our efforts to develop sense and antisense oligonucleotide (FANSAO) based temporal tunability of the translational event involving the *Carnitine Acyl Carnitine Translocase* (CACT) gene (Gene ID: 5724426) since it has a pivotal role in the regulation and accumulation of lipids within the cell. We here describe the design of a nanobiosystem composed of gold nanoparticles decorated with sense and antisense oligonucleotides that act as an optical remote control, using an LED source, to switch gene expression and tune bioaccumulation of lipids within *Chlamydomonas reinhardtii*.

In the realm of elucidating biological gene function, several traditional approaches have been employed, including the study of phenotypic manifestations resultant from targeted gene mutations or the utilization of using genome sequencing techniques for the identification of mutations. Nevertheless, these methodologies exhibit limitations in terms of expeditious gene function decipherment. Alternative, RNAi technology has demonstrated substantial efficacy, in the accelerated analysis of gene function across extensive gene pools within a diverse array of organisms [7]. Despite its advantages, RNAi technology is contingent upon the RNA-induced silencing complex (RISC), presenting constraints such as a truncated half-life and the intricate delivery of RNAi molecules [8]. Additionally, these methodologies lack the capacity for remote spatio-temporal modulation of gene interference within living plant cellular systems. Precise regulation of spatio-temporal gene expression or deletion is critical for research studies focused on elucidating gene function in biological systems [9, 10]. Accurate control of the gene silencing process in living plant cells is not only highly demanded to study molecular cell biology [11], cellular signaling pathways [12], and an assortment of synthetic biology applications [13] but it is also integral to the expression of several key endogenous genes in plant cells. The regulation of these endogenous genes is essential for ensuring proper development during the early stages of plant cell life, as well as for the controlled downregulation of the genes at specific temporal junctures. In light of the fact that conventional gene-interfering techniques do not offer precise spatio-temporal control of gene expression in living plant cells, we propose addressing this challenge by developing suitable vectors, such as nanocarriers and nanobiosystems, to facilitate the regulation of gene expression in plant cellular systems.

**Scheme 1:**
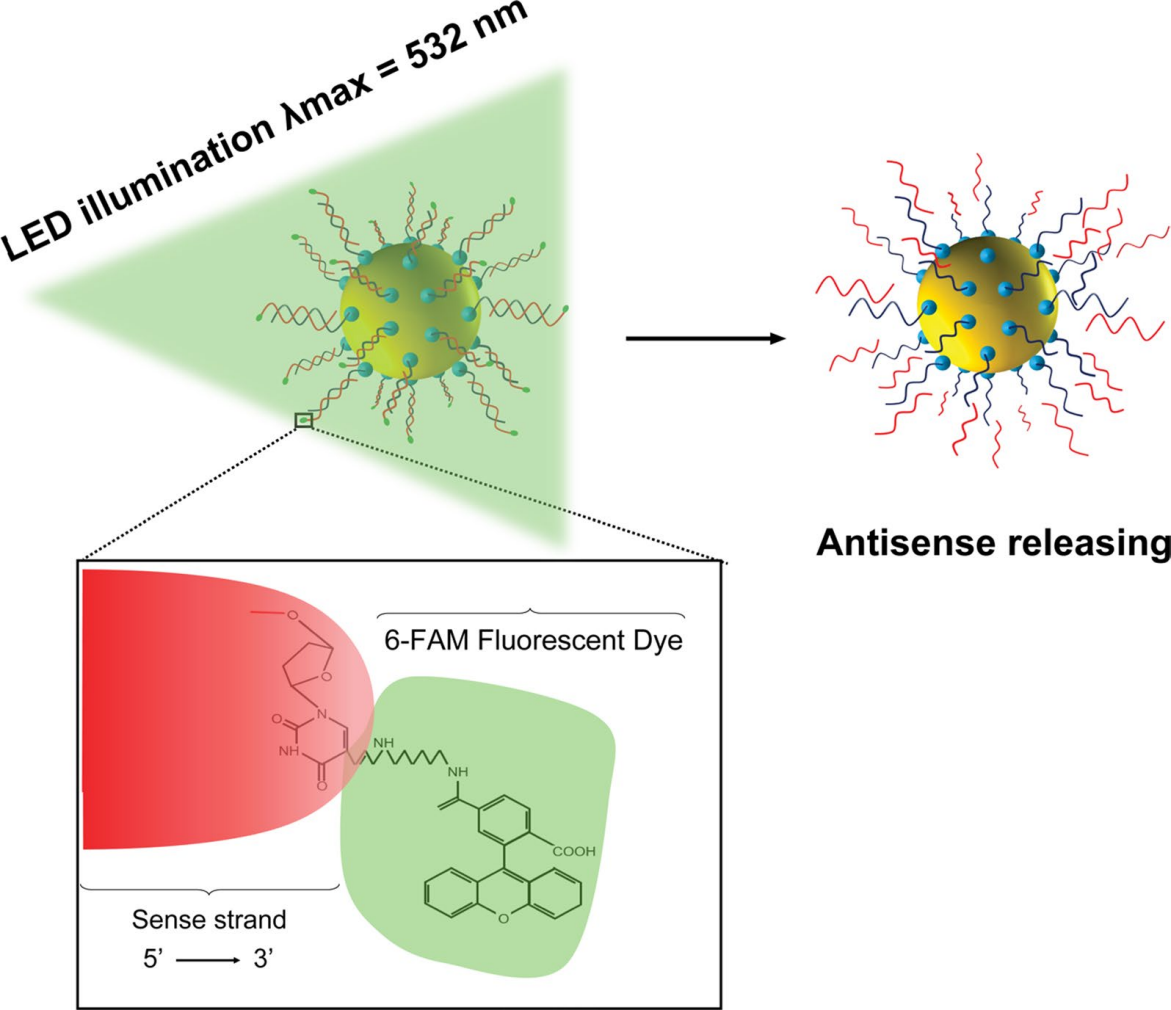
Photothermal release of fluorescently labeled DNA from FANSAO using LED illumination

Nanotechnology encompasses the study of materials exhibiting at least one dimension measuring less than 100 nm [14]. Nanomaterials possess specific physical and chemical properties [15], different than those expressed in the bulk due precisely to their scale. These unique characteristics render nanomaterials as valuable tools with fundamentally novel properties and a plethora of applications, including the delivery of biomolecules into plant systems, a highly desirable trait for the present study [16]. We proposed developing a platform technology endowed with spatio-temporal control over gene interference in plant cells, comprising gene delivery vehicles possessing the following core capabilities [17]: (1) Ability to selectively deliver biomolecules, including DNA and RNA; (2) protection of DNA and RNA from enzymatic dependent degradation while en-route, and (3) incorporation of mechanisms to release genetic material ‘on command’ upon reaching the site of interest. In light of these requirements, we proposed incorporating spherical gold nanoparticles (AuNPs) into our system due to their size and shape-dependent optical, electrical, and thermal properties, easily-tunable optical properties, known surface chemistry, and relatively easy synthesis. Spherical AuNPs exhibit unique optical absorptions and an extensive absorption cross-section attributable to their geometric nature. Most importantly, they can absorb optical energy at a specific wavelength, thereby providing high photothermal conversion efficiencies [18]. These exceptional optical properties render AuNPs as excellent candidates for various applications. Furthermore, the surface of the AuNPs can be readily conjugated with a variety of ligands with desired functional groups, such as thiol and amine [19]. Consequently, in this study, we used functionalized AuNP with sense and antisense oligonucleotides (FANSAO) as an optical switch to design a remotely controlled gene interference system in photosynthetic cells (Scheme 1). Our results demonstrate that through the delivery of FANSAO, the transcription of an endogenous gene of interest can be conditionally controlled. As depicted in our Scheme 1, the gene of interest is continuously expressed; however, it can be silenced upon exposure to an LED light source, where FANSAO receives the light, and as a result, this event leads to exciting the plasmon of the AuNPs (at the core part of FANSAO) and because of the high-energy decay of the excited plasmon through a non-radiative process, the temperature at the surroundings of the FANSAO is elevated. Subsequently, this phenomenon triggers the release of the antisense into the cellular cytosol. Moreover, switching off the LED light exposure halts the photothermal process, thereby stopping gene silencing and permitting the restoration of gene expression.

The use of lasers as a light source constitutes a conventional tool in photothermal therapies for biological systems. Nevertheless, lasers exhibit several disadvantages, including their elevated cost and the risk for thermal damage [20]. Consequently, in the present study, we sought to explore the use of light-emitting diodes (LED) as an alternative light source, given their novel, cost-effective and harmless nature. LEDs also present the advantages of being switchable, non-destructive for living organisms such as plant cells, and especially scalable for larger applications. In this work, we report our efforts to develop FANSAO-based temporal tunability of the translational event involving the *Carnitine Acyl Carnitine Translocase* (CACT) gene (Gene ID:5,724,426). The CACT is a member of the mitochondrial carrier family and is involved in fatty acid metabolism in *Chlamydomonas reinhardtii*, a unicellular plant model organism [21]. The selection of CACT gene regulation was predicated upon its pivotal role in the catabolism of long-chain fatty acids. Long-chain fatty acids cannot cross the outer and inner membranes of the mitochondria for β-oxidation without assistance. The fatty acid is first converted to acyl-COA, and then it is attached to carnitine to form the acyl-carnitine complex, which can enter the mitochondria. The CACT protein transports the acyl-carnitine complex into the matrix of the mitochondria. Carnitine is then removed from the long-chain fatty acid and transported back out of mitochondria by the CACT protein. CACT silencing prevents the shuttle-like action of carnitine from assisting fatty acids across the mitochondrial membranes, leading to a decreased fatty acid catabolism. This results in an increase in the lipid content within cells. Altogether, the results reported in this work demonstrate that the designed nanobiosystem is a platform technology with a myriad of applications, extending beyond the silencing of the CACT gene, which leads to an increased accumulation of lipids within plant cells.

## Results and discussion

### Identification of microalgae growth kinetics

The kinetic growth curve of microalgae can aid not only in the understanding of the kinetic models of microalgae growth to optimize cultivation conditions but can also provide a limited understanding of the relationship between algal growth and the storage and production of molecules, such as lipid compounds [22] which are one of the most important products of microalgae. We initially grew *C. reinhardtii* under specific conditions to determine its photoheterotrophic growth kinetics. *C. reinhardtii* is a single-cell green alga; because of its remarkable adaptability to different environments and short generation time, it is considered a model organism for various studies, such as those related to lipid metabolism [23]. The sequencing of its whole genome and the availability of several molecular research tools allowing genetic studies make this organism an attractive model system to study at the molecular level [24]. Photoheterotrophic growth of microalgae is one of the standard options many researchers use since recent mathematical studies indicated that the cultivation of microalgae under photoheterotrophic conditions leads to the (Scheme 1) accumulation of significant amounts of intracellular oil droplets (triacylglycerol-TAG) compared to other growth conditions such as photoautotrophic growth [25]. Hence, a photoheterotrophic culture was prepared for this experiment, and the samples were kept in a shaking incubator (LSI-3016R) while their optical densities (OD_680_) were measured. As shown in Additional file 1: Figure S1, photoheterotrophic growth curves demonstrate that the algal cells undergo logarithmic growth within the first four days and then slowly transition to their stationary phase. The sampling times were chosen from the kinetic growth curve and determined to be optimal shortly before the end of the exponential phase.

### AuNPs characterization

**Fig. 1:**
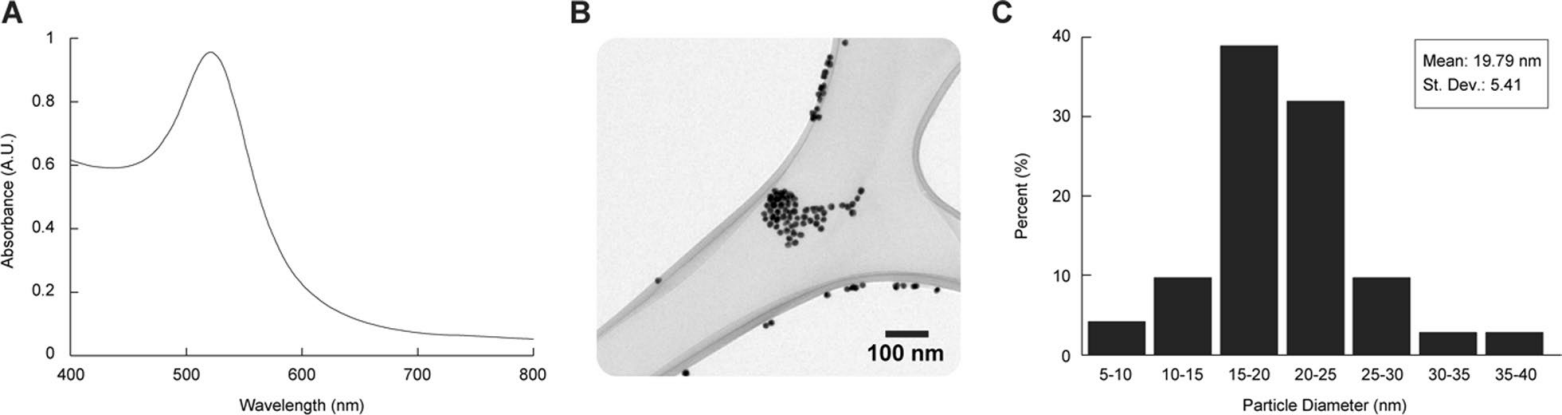
Characterization of the synthesized spherical AuNPs. **A** Surface plasmon absorption of AuNPs with a maximum absorbance at 521 nm. **B** TEM image of the synthesized AuNPs. **C** Particle size distribution obtained from TEM images

Next, we synthesized AuNPs, which would serve as antisense oligonucleotide carriers and gene expression regulators. The synthesis was performed through a one-phase synthesis method where AuNPs were prepared by reducing gold chloride salt (AuCl_3_) with trisodium citrate. The UV–vis spectrum of the reaction sample was recorded at the final reaction time point (4 h) (Fig. 1A). Figure 1A shows the 521 nm narrow surface plasmon resonance (SPR) peak, which correlates to the presence of gold nanoparticles in the solution. Moreover, this absorbance peak fits with the LED light wavelength (520–525 nm) to be used in this work, which would lead to a high-efficiency conversion of light to heat. The narrow peak indicates a homogenous size distribution of the AuNPs and the lack of agglomerates, thus confirming the stability and monodispersity of the synthesized AuNPs [26]. In addition, TEM was used to determine the shape, average size, and particle size distribution of the synthesized AuNPs. The TEM micrographs show that the synthesized particles are spherical (Fig. 1B) with a homogeneous size distribution (Fig. 1C). The particle distribution analysis determined the average size of the synthesized AuNPs to be 19.79 nm with a StDev of 5.41 nm (Fig. 1C). Nanoparticles with small diameter and uniform size distribution contribute to high physical and chemical performances for practical applications due to their large surface-to-volume ratio [27].

### Heat generation from the surface of the AuNPs

To explore the photothermal energy conversion displayed by AuNPs, in the biological system of microalgal cells, it was necessary to identify the behavioral pattern of heat generation when AuNPs were exposed to a specific light wavelength. Four factors: concentration and volume of AuNP solution, time illumination with LED, and distance of AuNP container from the LED light source, were evaluated using a central composite design approach-based response surface methodology analysis. The regression analysis showed which factors were statistically influential in the heat generation from the surface of the AuNPs (Additional file 1: Table S1). These significant variables are included in the final regression model.

Based on the surface response experiments, the mathematical relationship between the temperature change and the four influential factors was fitted by the following second-order polynomial model (Eq. 1):

1 $$Y\,=\,19.42+1.39 {x}_{1}-1.66 {x}_{2}-16.3{x}_{3}+3.18{x}_{4}+1.13{x}_{1}{x}_{2}+1.42{x}_{2}{x}_{3}-3.28{x}_{3}{x}_{4}-3.1 {{x}_{1}}^{2}-3.32{{x}_{2}}^{2}+8.67{{x}_{3}}^{2}-1.89 {{x}_{4}}^{2}$$

In the above equation, which is in terms of coded factors, Y represents the temperature change, × 1, × 2, × 3, and × 4 are the concentration of AuNPs solution, the volume of AuNPs solution, the distance of AuNPs container from LED, and illumination time, respectively.

An F test confirmed the obtained regression model, and the results showed that the model was highly significant (P-value < 0.0001), whereas its lack of fit was not significant (P value = 0.6178). Moreover, the regression model showed a high value for the correlation coefficient (99.78%), where an adjusted R^2^ (99.46%) and predicated R^2^ (98.13%) were used to evaluate the adequacy of the model fitting (Additional file 1: Table S1). The results demonstrate the close fit between the predicted and experimental values. Hence, it can be stated that there was a correct fit for the introduced quadratic model.

Further, we tested our regression model (Eq. 1) by comparing predicted temperature change values to those temperature values observed experimentally (Additional file 1: Figure S2A). As can be seen, the predictions are very close or match the fitted line, which reflects a very good fit for the quadratic model. In addition, the residuals versus the predicted response plot showed no clear pattern or trend of the data, demonstrating that our model has sufficient adequacy (Additional file 1: Figure S2B). The regression model obtained is a foundation for predicting and optimizing the effective variables and obtaining a specific temperature change of interest.

### Construction of switches through the conjugation of sense and antisense oligonucleotides to AuNPs

**Fig. 2:**
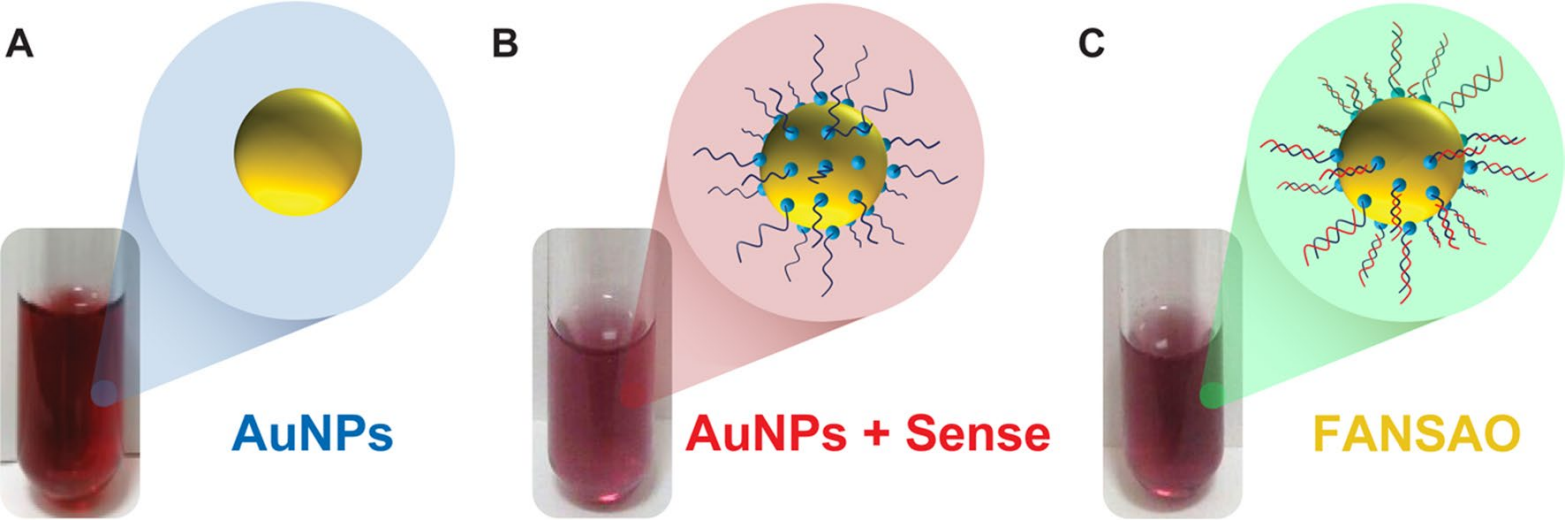
Color change of AuNPs solution in the FANSAO synthesis process. **A** Pure AuNPs. **B** Conjugation of AuNPs to sense oligonucleotides. **C** Conjugation of antisense oligonucleotides to AuNPs + Sense oligonucleotides

**Fig. 3:**
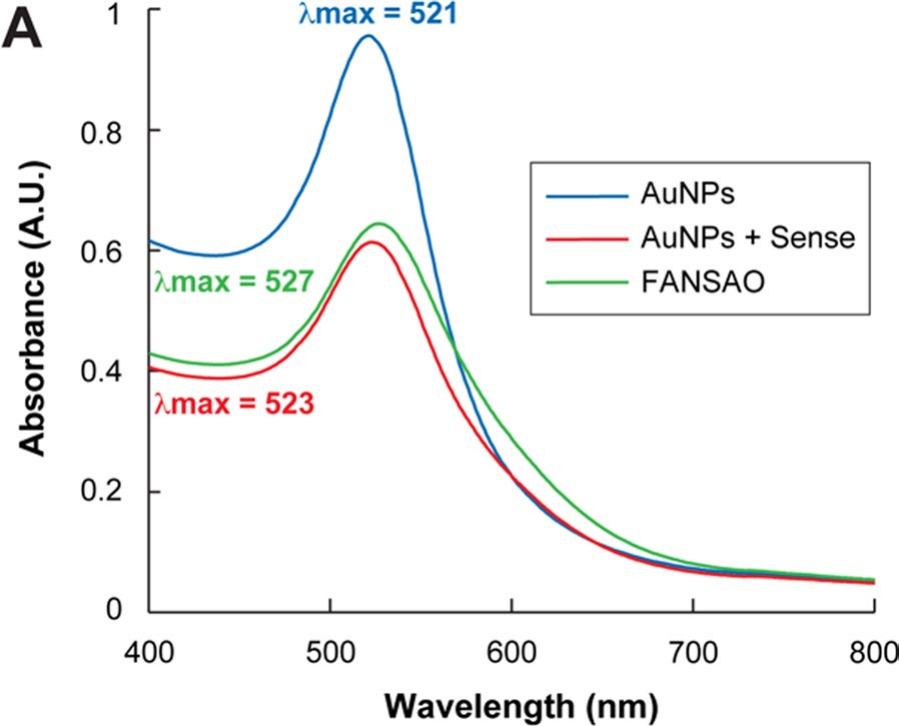
UV- vis absorption spectrum changes of AuNPs solution in the FANSAO synthesis process. Changes in the UV–vis absorption spectrum when an aqueous dispersion of 19.79 nm AuNPs is treated with thiolated sense oligonucleotides and then with the antisense oligonucleotides, showing a decrease in absorption rate and a slight red shift of the SPR band (from λ_max_ = 521 nm—λ_max_ = 527 nm) indicative of ligand exchange [17]

The high surface area exposed in AuNPs facilitates surface modification for remote-controlled release and precise delivery of coupled interfering agents [28]. Therefore, our synthesized spherical gold nanoparticles were used to conjugate sense and antisense oligonucleotides. The surface chemistry of AuNPs permits binding with thiol groups through covalent bonding. Therefore ss-DNA that had been chemically modified to include an SH-group was simply conjugated to the AuNPs surface. Covalent attachment of nucleic acids to AuNPs is an effective means of transporting post-transcriptional gene-silencing oligonucleotides, where the modification cannot inhibit biological activity [29]. Hence, a certain amount of modified sense oligonucleotides was added to a prepared AuNPs solution (Fig. 2A–C) which initially exhibited an absorbance peak at 521 nm (Fig. 3). After incubating the AuNPs solution on a rocker at room temperature with the modified sense oligonucleotides, UV–vis absorbance spectroscopy changed, confirming the 15-mer 3’-thiolated sense strands attachment (Fig. 3). Next, the AuNPs decorated with the 15-mer 3’-thiolated sense strands were hybridized with their conjugates to complement and incorporate the fluorescence*-*labeled antisense (15-mer 3’-(6-FAM)) strands. As can be observed in Fig. 2A–C, replacing the citrate capping ligand of the original AuNPs with the thiolated oligonucleotides leads to a color change from dark to pale red. Moreover, the initial thiolated sense oligonucleotide incorporation into the surface of the AuNPs leads to a reduction in the maximum absorbance peak and a small but observable redshift [17, 30, 31] of the nanoparticle’s SPR band from 521 to 523 nm (Fig. 3). Finally, when the antisense oligonucleotides were hybridized to the sense strands, and the final structure of FANSAO was accomplished, an additional redshift from 523 to 527 nm was observed (Fig. 3). The dense shell on the AuNPs composed of the double strands of oligonucleotides on the surface of AuNPs could provide additional stability for AuNPs [32] and effectively prevent the occurrence of degradation by nucleases, protecting the load delivered to the cell [33].

### *In-vitro* confirmation of antisense photothermal dehybridization

**Fig. 4:**
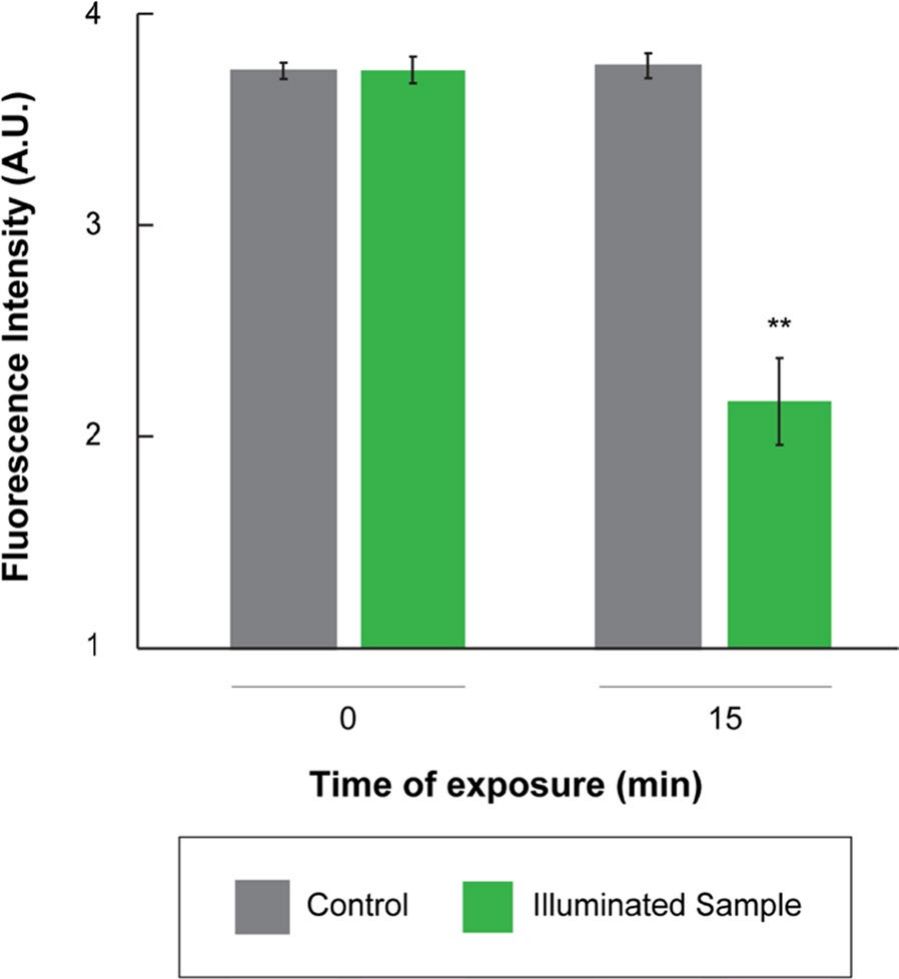
Photothermal dehybridization of 15 bp oligonucleotides with known melting temperatures (54.9 ºC). Antisense oligonucleotides are 6-FAM-labeled

The oligonucleotide (15 bp) attached to the AuNPs exhibits a known melting temperature (Tm) (54.9 $\sim$ 55 ºC). The antisense oligonucleotides are released from the sense strand at the melting temperature. This means that our 200 µL solution containing the FANSAO (with a length of 15 bp) attached to the AuNPs needed to be illuminated under specific parameters to achieve the temperature increase from the cultivation temperature to the Tm to deliver the antisense oligonucleotide and observe the gene silencing phenotype in our biological system. Therefore, using the developed regression model (Eq. 1), the appropriate concentration of AuNP solution, volume, time illumination of light, and distance of AuNP container from the light source, were determined to achieve the required experimental temperature increments to the Tm. As observed in Fig. 4, when the temperature on the FANSAO reaches the melting temperature of the double-stranded oligonucleotide, the fluorometer measurements showed that the fluorescent intensity decreases sharply. These results indicate that DNA-based antisense oligonucleotide has been released into the solution [34]. In addition, we demonstrate that Eq. 1 can be used to optimize experimental conditions and achieve the required temperature increases with exposure to a light source. These *in-vitro* test results were a prelude to testing our nanobiosystem *in-vivo* in microalgae cells.

### *In-vivo* antisense photothermal dehybridization in microalgae cells and RNA quantification

**Fig. 5:**
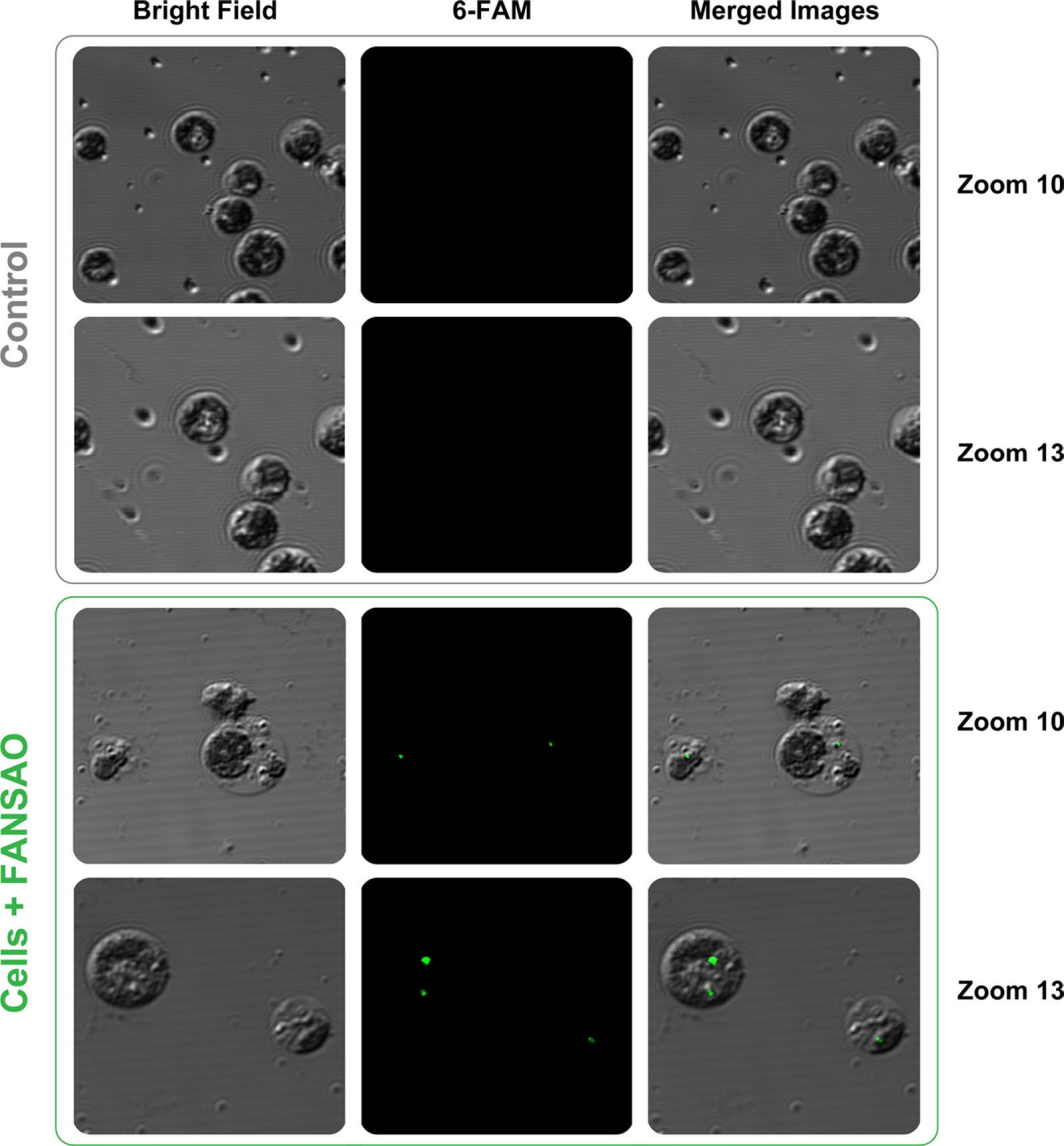
Confocal microscopy images; dsDNA-functionalized AuNPs successfully delivered into the microalgae cells

*C. reinhardtii* microalgae strains were subcultured until they reached the early exponential phase with a more than 2 × 10^6^ cell/ml cell density. Next, the cells were centrifuged and resuspended at a cell density of 25 × 10^7^ cells/ml. 400 µl of microalgal cells were mixed with 800 µL of FANSAO attached to AuNPs in 1.5 ml microtubes and electroporated for 30 ms. After incubation for 15 min and recovery for 12–16 h in the dark at 25 °C, the cells were harvested and resuspended in a 400 μl TAP medium. Confocal microscopy images of our samples show that the control algae do not present fluorescence. In contrast, the FANSAO-treated samples show internalization of the dsDNA-functionalized AuNPs into the microalgae cells after 14 h in both zoom images (Fig. 5).

**Fig. 6:**
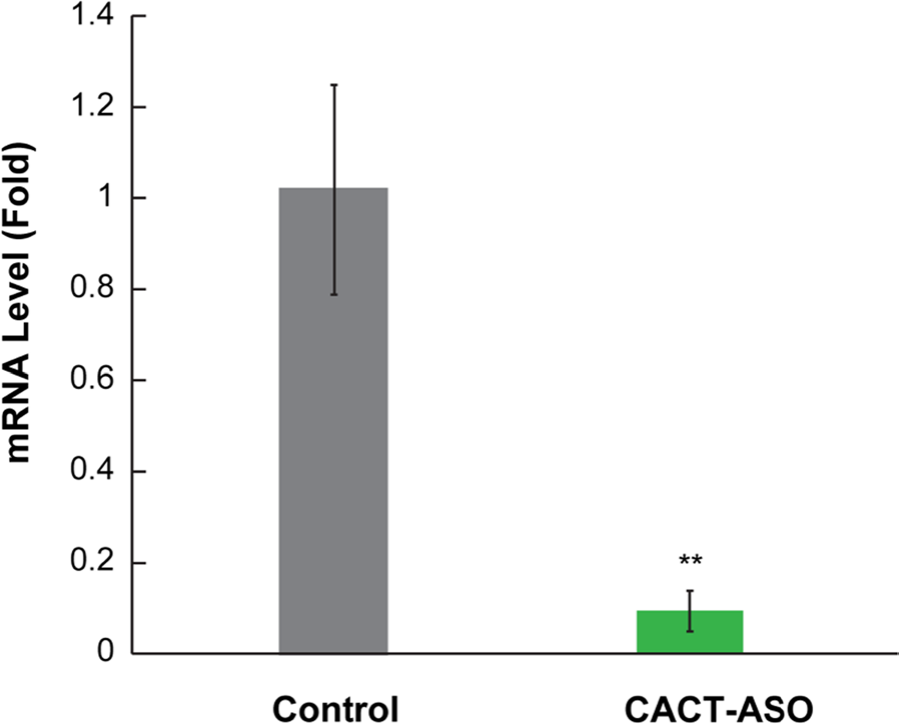
comparative RT-PCR; The mRNA abundance of the CACT gene in *C. reinhardtii* cells that received FANSAO; The expression of the CACT gene in comparison to control revealed decreased expression of this gene at the time that the cells illuminated by the LED light source to gene silencing. Data are expressed as mean ± SEM; n = 3 well for each group; **P $<$ 0.01 vs. control (non-treated dsDNA-functionalized AuNPs) cells

Next, we evaluated the dehybridization of the sense-antisense oligonucleotides *in-vivo*, which would lead to gene silencing by measuring the abundance of target gene-specific mRNA by comparative RT-PCR in microalgae cells that received the dsDNA-functionalized AuNPs. The results revealed a significant difference in mRNA expression levels between the control and the treated groups. As shown in Fig. 6, the CACT mRNA abundance decreased up to 90.7% compared to control samples that didn’t receive any FANSAO, indicating high-efficiency silencing by this method.

This result confirmed that LED light sources could heat the surface of AuNPs to the melting temperature of the sense and the antisense oligonucleotides that were located on the NPs. The temperature increase, as observed *in-vitro*, led to the release of the antisense oligonucleotides, which were able to bind to the start codon section of the CACT mature mRNA. Afterward, the mRNA/antisense DNA heteroduplex was formed and was recognized and degraded by cytosolic RNase H enzymes, thereby achieving significant silencing of the CACT gene at the required time (on-demand silencing). It should be noted that because of the strong gold-thiol covalent bonds, the surface of the AuNPs remained functionalized with the thiolated complementary sense strands (the antisense oligonucleotides just released from their complementary strands) after illumination. As mentioned previously, this is important and hypothesized since the AuNPs kept showing no cytotoxicity effects, even though there are reports that demonstrate bare gold nanoparticles can interact with proteins and induce misfolding under physiological conditions, leading to cell death [35].

**Fig. 7:**
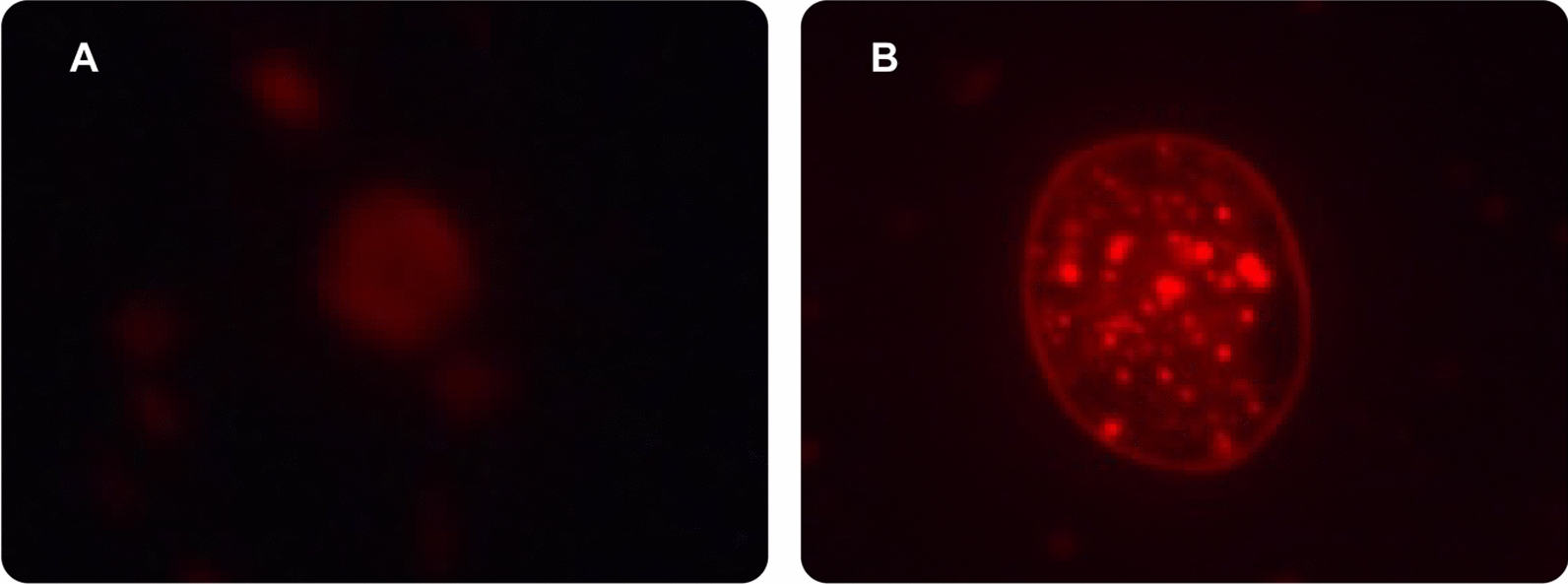
Fluorescence images of Nile red-stained *C. reinhardtii* cells, 4 h after gene silencing; **A** Control sample contains cells that didn’t receive any FANSAO. **B** Cells that received FANSAO contain considerable amounts of red fluorescent dots related to lipid bodies

**Fig. 8:**
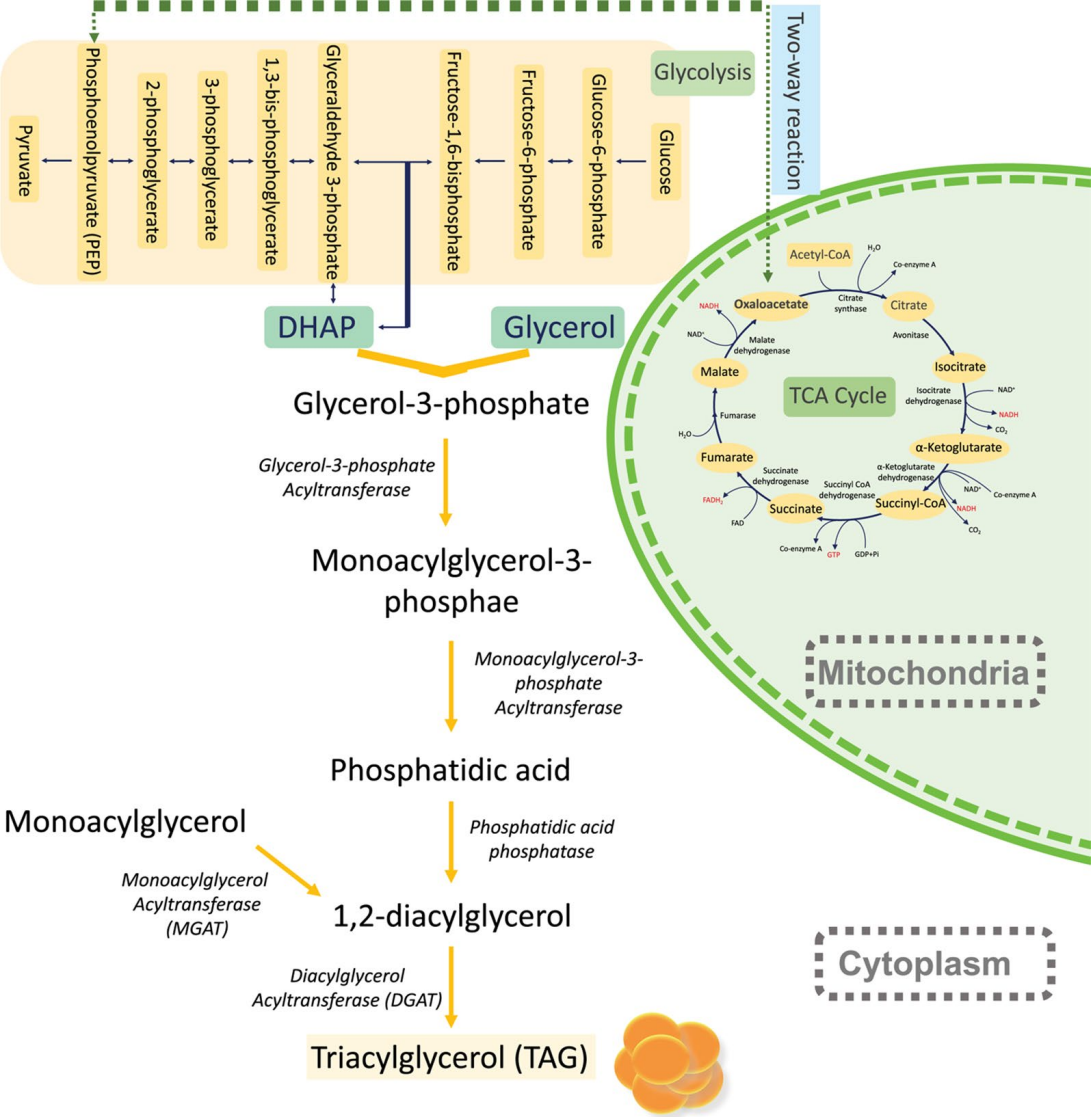
TAG synthesis pathway; Stopping of Krebs cycle due to lack of Acetyl-CoA and consequently conversion of increased Oxaloacetate to PEP. Next, the synthesis of the TAG from DHAP is one of the intermediate compounds of the Glycolysis pathway

To confirm the CACT gene silencing effect on cellular lipid accumulation, treated and non-treated *C. reinhardtii* cells were stained with Nile red fluorescent dye and visualized using fluorescence microscopy. As shown in our results, Nile red staining of lipid bodies confirmed a substantial accumulation of lipids within the treated samples (Fig. 7B) compared to the untreated control (Fig. 7A). A suggested explanation of the increased lipid bodies in the samples with the silenced CACT gene can be illustrated in Fig. 8. Silencing the CACT gene leads to a decrease in CACT protein synthesis, affecting the transfer of fatty acids into the matrix of the mitochondria and accumulating them within the cell. Moreover, other biochemical pathways can also lead to the accumulation of fatty acids due to CACT gene silencing. Since the fatty acids cannot be broken through the Beta-oxidation cycle, which takes place within the mitochondria, there is a decrease in the production of Acetyl-CoA, an essential ingredient for the Krebs cycle. Therefore, decreases in Acetyl-CoA can disrupt the Acetyl-CoA and Oxaloacetate balance within the cells. Extra amounts of Oxaloacetate, instead of participating in the TCA cycle, during a two-way reaction, could be converted to Phosphoenolpyruvate (Fig. 8). In this step, Phosphoenolpyruvate, during several two-way reactions, could be converted to Dihydroxyacetone phosphate. Eventually, the produced Dihydroxyacetone phosphate combined with Glycerol could be converted to TAG (Fig. 8). The activation of both of these pathways, due to CACT gene silencing, could be a possible explanation for the substantial accumulation of lipids observed in Fig. 7B.

## Conclusion

The augmentation of the multidisciplinary nature of scientific research is poised to play a pivotal role in addressing numerous challenges in plant biotechnology. One such challenge is the precise control of spatio-temporal gene expression in living plant cells, which currently represents a bottleneck for efficacious plant genetic engineering. In the present study, we report the utilization of FANSAO for spatio-temporally controlled gene silencing in plant cells by coupling them with AuNPs to harness their opto-thermal energy conversion properties. Although previous reports have shown the potential toxicity of certain nanoparticles (Ag, CuO, ZnO, Se, Pd, FeO, and especially AuNPs) to some organisms at high concentrations [36–41], our data show that functionalizing AuNPs with thiolated complementary sense strands, eliminates such toxic effects against microalgae cells. Furthermore, we demonstrated the feasibility of using LED as a light source to stimulate AuNPs and induce photothermal dehybridization of oligonucleotides within plant cells, thereby showcasing the potential for large-scale implementation, in contrast, to laser-based approaches. The results reported in this manuscript showed that antisense oligonucleotides from FANSAO can be remotely light-triggered and released both in vitro and in vivo within microalgae. Subsequently, the released antisense oligonucleotides can induce CACT gene silencing up to 90.7%, culminating in the accumulation of lipid bodies within microalgae cells. The methodology platform reported here constitutes a promising strategy for spatio-temporal gene silencing in plant cells, such as microalgae, with diverse applications in bioprocess engineering and fundamental studies of gene roles in biological pathways. Moreover, we have demonstrated that our platform possesses direct relevance to the energy sector since our microalgal cells exhibited increased bioproduction of fatty acids, which serve as main precursors for biofuels.

## Methods

### Synthesis and characterization of the AuNPs

Water-soluble AuNPs were synthesized via the Turkevich et al*.* method reported in the literature [15, 42]. First, 50 ml of 0.5 mM HAuCl_4_ solution was prepared in a beaker and boiled on a magnetic stirrer until it was fully dissolved and the solution started to steam. At the boiling point, 5 ml of 38.8 mM trisodium citrate solution was added to the beaker, and the resulting solution instantly began to turn to a maroon color. After reacting for 10 min at boiling temperatures, the reaction mixture was allowed to cool slowly by turning the heating of the hot plate off, halted stirring, and leaving it at room temperature. With time, the solution shifted from a maroon color to red once it reached room temperature without stirring. The synthesized AuNPs were characterized by transmission electron microscopy (TEM, FEI-TITAN) and UV-vis spectroscopy in a spectrophotometer (Multiskan™ GO).

### Microalgae cultivation and maintenance

We used C. reinhardtii (ATCC^®^ PRA­142™), the CC­503 cw92 mt + strain, in this work. For the culturing of the *C. reinhardtii* stocks, 800 ml cylindrical glasses were used. The initial amount of the algae inoculum was 5% of the total medium volume. Cultures were grown in TAP-medium (Gibco) in a standard shaking incubator (LSI-3016R) with a shaking speed of 140 rpm at a temperature of 25 °C and a light intensity of 60 μmol/m^2^/s (under alternate 14 h light and 10 h dark cycles). The light-scattering properties of *C. reinhardtii* cells are used to measure the algal culture's optical density (OD), which correlates linearly to the algal cell density. A 1 ml sample of *C. reinhardtii* was extracted daily from the culture and analyzed under the appropriate wavelength (680 nm), where *C. reinhardtii* showed the highest peak for chlorophyll. The experiments were repeated three times, and curves of cell density for OD_680_ were drawn.

### Determination of the temperature profile of AuNPs

To determine the behavioral pattern of temperature increases in AuNPs surfaces when exposed to green light, an LED with a peak wavelength of 520–525 nm was employed. In this regard, statistical approaches were used, and four factors were investigated by a central composite design approach-based response surface methodology analysis. The variables consisted of concentration and volume of AuNP solution, time illumination of light, and distance of AuNP container from the LED. They were evaluated by a set of 25 experiments at different levels. It is noteworthy that Design Expert software (version 11.0.5, STAT-EASE, Minneapolis, MN, USA) was used to design and analyze the experimental data.

### Attachment of DNA to AuNPs to make FANSAO

First, the disulfide bonds of thiol-modified oligonucleotides were reduced with DDT (Thermo fisher science R0862) to achieve the active sulfhydryl form. Then, the activated sulfhydryl forms of oligonucleotides were purified using NAP10 purification columns (GE Healthcare). To conjugate sense oligonucleotides to the surface of the AuNPs, 22 μL of 100 μM thiolated sense oligonucleotides were incubated with 40 μL of PBS (pH 7.4) and 2000 μL of AuNPs (6.52 × 10^11^ particles/ml or 1.08 nM) on a rocker for 8 h. The sense oligonucleotides were attached to the surface of the AuNPs through the thiol (-SH) group on the 3’ end.

To hybridize antisense oligonucleotides to the sense oligonucleotides, 22 μL of 100 μM antisense oligonucleotides were added to the AuNPs-sense solution. This mixture was then heated for 2 min at 80˚C and heated for 15 min at 65˚C. The mixture was finally incubated at room temperature on a rocker for 8 h to ensure maximum hybridization.

### *In-vitro* test of antisense photothermal dehybridization

Photothermally generated heat in the surface of the nanoparticles when exposed to the LED would lead to antisense releasing and gene silencing in living microalgae cells. To validate this, we first confirmed in vitro the photothermal dehybridization of antisense strands from the FANSAO by using an oligonucleotide (15 bp) with known melting temperatures (54.9 $\sim$ 55 ℃): (15 bp sense) 5′- ACACCTTATGGAGCC-3′-(CH_2_)_6_-thiol, (15 bp antisense) 5′-GGC TCCATAAGGTGT -3′-6-FAM. As expressed before, we identified effective variables for increasing the temperature of the solution containing AuNPs using statistical approaches. To provide the desired temperature for antisense releasing ($\sim$ 55 ℃), based on statistical analysis and final optimization by response surface method, we illuminated 200 µL FANSAO for almost 15 min. The fluorescence intensity of the FANSAO was measured by Microplate Readers (Fluoroskan™ Microplate Fluorometer).

### FANSAO delivery into the microalgae cells

Generally, the import of exogenous DNA into the unicellular, green alga *C. reinhardtii* is limited by their rigid cell wall. Although various methods, such as glass beads agitation, electroporation, and microparticle bombardment, have been successfully used [43–48] to import DNA into the plant cells such as unicellular microalgae, there are no successful reports in the literature on ssDNA or dsDNA-functionalized AuNPs (DNA-AuNPs) delivery into the microalgae cells. However, we imported the FANSAO into the *C. reinhardtii* cells by electroporation following the protocol from Invitrogen using GeneArt^®^ MAX Efficiency^®^ reagent. Preparation of the sample was performed according to the manufacturer’s instructions with some modifications. The essential modifications included the sample type (FANSAO instead of linear DNA or plasmid) and the type of electroporation device (Eppendorf Eporator^®^), DNA concentration, and sample size.

Microalgae strains were subcultured in 200 ml of TAP medium until they reached the early exponential phase and reached a cell density of more than 2 × 10^6^ cell/ml (the total amount was 45 × 10^7^ cells in 200 mL of TAP medium). The cells were then harvested by centrifugation (for 2500 rpm and 5 min) followed by washing twice with 10 mL GeneArt^®^ MAX Efficiency^®^ Transformation Reagent. Afterward, they were resuspended at a cell density of 25 × 10^7^ cells/ml, and 400 µl microalgal cells were mixed with 800 µL FANSAO in a 1.5 ml microtubes and then divided into ice-colds 0.2 cm cuvette for electroporation process (almost 30 ms). This was followed by electroporation using the Eppendorf Eporator^®^ electroporation system at 500 V and 600Ω. They incubated on the bench for 15 min and then transferred to 10 ml TAP-40 mM sucrose medium. They were allowed to recover for 12–16 h in the dark at 25 °C and shook at low speed. Then cells were harvested and resuspended in a 400 μl TAP medium. (confirmation of FANSAO delivery was performed by confocal microscopy).

### Experimental setup for photothermal releasing of antisense oligonucleotides for gene silencing

After delivering the FANSAO into the microalgae cells, the green LED device (with a peak wavelength of 520–525 nm and a viewing angle of $140^\circ$) was positioned in front of the culture. The green LED chip was used to illuminate the 400 µl microalgae cells containing FANSAO for almost 6 min to reach the surrounding temperature of AuNPs surface up to the Tm of the sense and antisense oligonucleotides (Tm=$\sim$ 55ºC), which were conjugated on the AuNPs surfaces. Then, samples in three repetitions were incubated at 25 °C (normal growth temperature of *C. reinhardtii* strain) for 2.5 h. In this experiment, we also prepared control samples with cells that had not received any FANSAO and had not been exposed to any illumination. Afterward, the samples were incubated at 25 °C for 2.5 h (this experiment was carried out with three repetitions).

### RNA extraction and quantification

RNA extraction was performed with an Invitrogen™ TRIzol™ reagent (Cat. No. 15596026) from Invitrogen following the protocol in the technical manual. RNA concentrations were measured using a Shimadzu BioSpec-Nano Micro-Volume Spectrophotometer. Then, DNase treatment was performed using DNase I, an RNase-free kit from Invitrogen™ (Cat. No. MAN0012000), following the protocol in the technical manual to remove genomic DNA from the RNA preparation. Generation of cDNA was performed using SuperScript™ IV First-Strand Synthesis System from Invitrogen™ (Cat. No. 18091050), following the protocol in the technical manual. Gene-specific primers were designed to amplify approximately 157–263 bp fragments in length. For the quantification of gene expression, Real-time PCR (RT-PCR) was carried out on AriaMx Real-time PCR System using the SYBR Green dye; innuMIX Real-time PCR DSGreen Standard (Analytic Jena). RT-PCR reactions were carried out in a total volume of 20 µl, with 10 µl mentioned SYBR Green PCR Master Mix, 1 µl of a 5 µM primer of each one of forward and reverse primers, 100 ng of cDNA as template, and sterilized Milli-Q water up to 20 µl. RT-PCR program is shown in Additional file 1: Table S2.

The alpha-tubulin 1 (TUA1) gene was an internal control for the quantification assays. For gene expression analysis by RT-PCR, the expression values were calculated according to the Livak method [49]. Additional file 1: Table S3 shows all primer sequences used in this work. It is worth mentioning that threshold values were determined manually and normalized between plates. All RT-PCRs were run in triplicate (also, this experiment had three biological repetitions). Finally, to enable further understanding of the CACT gene silencing effect on cellular lipid formation increase, *C. reinhardtii* cells were stained (4 h after silencing) with a Nile Red fluorescent dye (Sigma-Aldrich); microalgae cells (200 µl) were added with 50 µl of Nile Red dye (10 µg mL^‒1^ DMSO stock) and incubated for 10 min in the dark and room temperature followed by washing with sterilized Milli-Q water. Then, the slides with stained microalgae samples were prepared and observed under a fluorescence microscope (Leica DM500 microscopes) at 596 nm excitation.

## Supplementary Information

**Additional file 1: Figure S1.** Cell growth profile of C. reinhardtii in TAP growth medium. Error bars represent standard deviations from three independent growth experiments (Each point in the curves represents the mean of three replicates ± standard deviation (n = 3)). **Figure S2. (a) **The predicted versus the observed response values.** (b) **The residuals versus the observed response values. **Table S1.** Analysis of variance (ANOVA) for the response surface of the Quadratic model. **Table S2.** Thermocycler program (RT-PCR) is used to quantify gene expression. **Table S3.** Modified DNA oligonucleotides and their melting temperatures (Tm).
