# Supplementary material for: Design of a nanobiosystem with remote photothermal gene silencing in Chlamydomonas reinhardtii to increase lipid accumulation and production
Source: Microb Cell Fact. 2023 Mar 31;22:61. doi: 10.1186/s12934-023-02063-9 (PMC10064687; doi:10.1186/s12934-023-02063-9)
Supplement: Supplementary file 1 — Additional file 1: Figure S1. Cell growth profile of C. reinhardtii in TAP growth medium. Error bars represent standard deviations from three independent growth experiments (Each point in the curves represents the mean of three replicates ± standard deviation (n = 3)). Figure S2. (a) The predicted versus the observed response values. (b) The residuals versus the observed response values. Table S1. Analysis of variance (ANOVA) for the response surface of the Quadratic model. Table S2. Thermocycler program (RT-PCR) is used to quantify gene expression. Table S3. Modified DNA oligonucleotides and their melting temperatures (Tm). [file 12934_2023_2063_MOESM1_ESM.docx]

**Design of a NanoBioSystem with Remote Photothermal Gene Silencing in *Chlamydomonas reinhardtii* to Increase Lipid Accumulation and Production**

Hossein Alishah Aratboni^1,2, †^, Nahid Rafiei^1,2,3, †^, Ashanti Concepción Uscanga-Palomeque^4^, Itza Luna Cruz^4^, Roberto Parra-Saldivar^5^, Jose Ruben Morones-Ramirez^1,2^*

^1^ Universidad Autónoma de Nuevo León, UANL. Facultad de Ciencias Químicas, Av. Universidad s/n. CD. Universitaria, San Nicolás de los Garza 66455, Nuevo León, México.

^2^ Centro de Investigación en Biotecnología y Nanotecnología, Facultad de Ciencias Químicas, Universidad Autónoma de Nuevo León. Parque de Investigación e Innovación Tecnológica, Km. 10 autopista al Aeropuerto Internacional Mariano Escobedo, 66629 Apodaca, Nuevo León, México.

^3^ Department of Plant Production and Genetics, School of Agriculture, Shiraz University, Km. 12 Shiraz‑Isfahan highway, Bajgah area, 71441‑65186, Shiraz, Iran.

^4^ Universidad Autónoma de Nuevo León, UANL. Facultad de Ciencias Biológicas, Av. Universidad s/n. CD. Universitaria, San Nicolás de los Garza 66455, Nuevo León, México.

^5^ ENCIT - Science, Engineering and Technology School, Tecnologico de Monterrey, Ave. Eugenio Garza Sada 2501, Monterrey, N.L., CP 64849, Mexico

* Correspondence: [jose.moronesrmr@uanl.edu.mx](mailto:jose.moronesrmr@uanl.edu.mx)

^†^ Hossein Alishah Aratboni and Nahid Rafiei contributed equally to the work

Supporting Information

**Figure S1** Cell growth profile of C. reinhardtii in TAP growth medium. Error bars represent standard deviations from three independent growth experiments (Each point in the curves represents the mean of three replicates ± standard deviation (n = 3)).

**
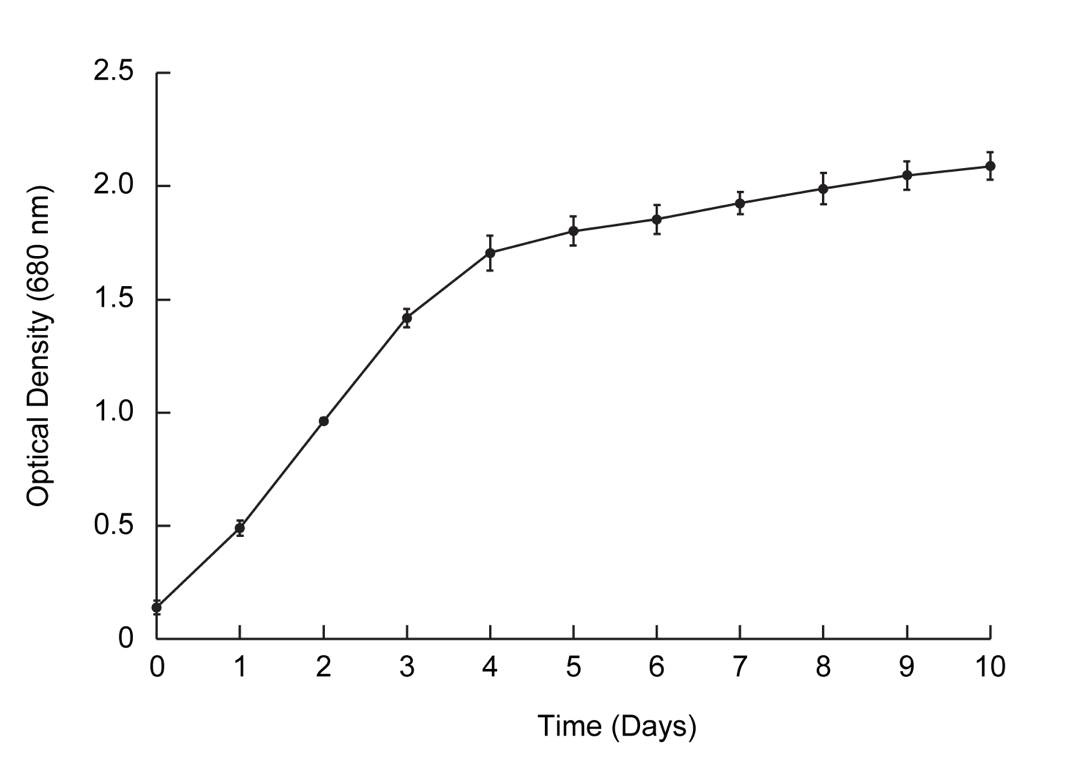
**

**Figure S2. (a)** The predicted versus the observed response values. **(b)** The residuals versus the observed response values.


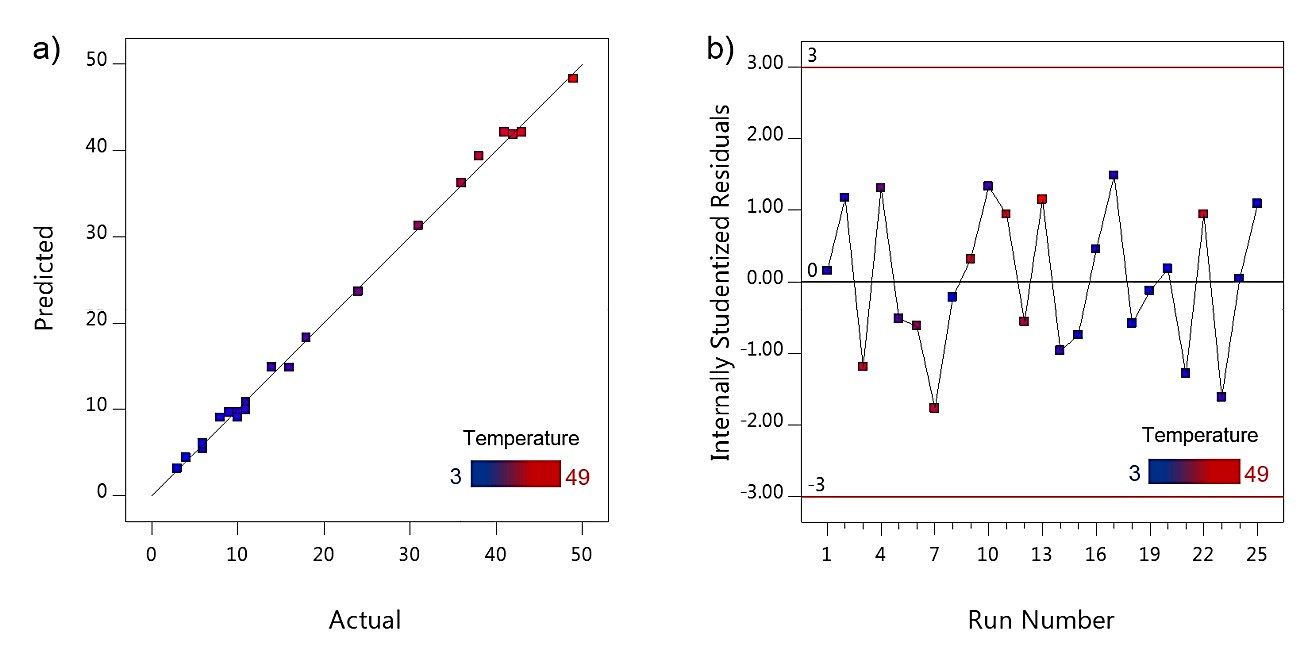


Additional file 1: Table S1 Analysis of variance (ANOVA) for the response surface of the Quadratic model

| Source | Sum of Squares | df | Mean Square | F-value | p-value |  |
| --- | --- | --- | --- | --- | --- | --- |
| Model | 5576.39 | 14 | 398.31 | 316.84 | < 0.0001 | significant |
| Concentration ($x_{1}$) | 23.22 | 1 | 23.22 | 18.47 | 0.0016 |  |
| Volume ($x_{2}$) | 39.77 | 1 | 39.77 | 31.64 | 0.0002 |  |
| Distance ($x_{3}$) | 3694.59 | 1 | 3694.59 | 2938.89 | < 0.0001 |  |
| Time ($x_{4}$) | 135.00 | 1 | 135.00 | 107.39 | < 0.0001 |  |
| $x_{1}x_{2}$ | 12.83 | 1 | 12.83 | 10.21 | 0.0096 |  |
| $x_{1}x_{3}$ | 2.40 | 1 | 2.40 | 1.91 | 0.1974 |  |
| $x_{1}x_{4}$ | 0.5598 | 1 | 0.5598 | 0.4453 | 0.5197 |  |
| $x_{2}x_{3}$ | 16.67 | 1 | 16.67 | 13.26 | 0.0045 |  |
| $x_{2}x_{4}$ | 1.44 | 1 | 1.44 | 1.14 | 0.3101 |  |
| $x_{3}x_{4}$ | 85.58 | 1 | 85.58 | 68.08 | < 0.0001 |  |
| ${x_{1}}^{2}$ | 17.88 | 1 | 17.88 | 14.22 | 0.0037 |  |
| ${x_{2}}^{2}$ | 43.96 | 1 | 43.96 | 34.97 | 0.0001 |  |
| ${x_{3}}^{2}$ | 383.06 | 1 | 383.06 | 304.71 | < 0.0001 |  |
| ${x_{4}}^{2}$ | 18.35 | 1 | 18.35 | 14.59 | 0.0034 |  |
| Residual | 12.57 | 10 | 1.26 |  |  |  |
| Lack of Fit | 5.40 | 5 | 1.08 | 0.7541 | 0.6178 | not significant |
| Pure Error | 7.17 | 5 | 1.43 |  |  |  |
| Cor Total | 5588.96 | 24 |  |  |  |  |
| Quality of quadratic model |  |  |  |  |  |  |
| Correlation coefficient (R2) |  |  | 0.9978 |  |  |  |
| Adjusted R2 |  |  | 0.9946 |  |  |  |
| Predicated R2 |  |  | 0.9813 |  |  |  |

P-values less than 0.05 indicate model terms are significant

Additional file 1: Table S2 Thermocycler program (RT-PCR) is used to quantify gene expression.

| Step | | Temperature | Time (s) | Cycles |
| --- | --- | --- | --- | --- |
| Hot Start | | 95ºC | 120 | 1 |
| Denaturation | | 95ºC | 15 | 40 |
| Annealing | | 60ºC | 30 |  |
| Extension | | 72ºC | 30 |  |
| Melt | Denaturation | 95ºC | 30 | 1 |
|  | Annealing | 65ºC | 30 |  |
|  | Extension | 95ºC | 30 |  |

Additional file 1: Table S3 Modified DNA oligonucleotides and their melting temperatures (Tm).

| Strand | Sequence (5’ to 3’) | Tm (ºC) | Modification | Length of production (bp) |
| --- | --- | --- | --- | --- |
| Sense (15 bp) | ACACCTTATGGAGCC | 54.9 | 3’-(CH_2_)_6_- thiol | - |
| Antisense (15 bp) | GGCTCCATAAGGTGT | 54.9 | 3’-(6-FAM) | - |
| F primer of CACT gene | CGAGGAACACCTTATGGAGCC | 64.6 | - | 263 |
| R primer of CACT gene | TGGCTGACTGTACTGAAGCG | 64.5 | - |  |
| F primer of alpha tubulin 1 (TUA1) gene | ATGGAGGAGGGTGAGTTCTC | 63.2 | - | 157 |
| R primer of alpha tubulin 1 (TUA1) gene | ACTACACTTGCTGCTACCCT | 63.5 | - |  |

NCBI accession numbers of CACT and TUA1 genes are XM_001698874.1 and XM_001691824.1, respectively.
